# P63 targeted deletion under the FOXN1 promoter disrupts pre-and post-natal thymus development, function and maintenance as well as induces severe hair loss

**DOI:** 10.1371/journal.pone.0261770

**Published:** 2022-01-25

**Authors:** Heather E. Stefanski, Yan Xing, Jemma Nicholls, Leslie Jonart, Emily Goren, Patricia A. Taylor, Alea A. Mills, Megan Riddle, John McGrath, Jakub Tolar, Georg A. Hollander, Bruce R. Blazar

**Affiliations:** 1 Division of Blood and Marrow Transplantation, Department of Pediatrics, University of Minnesota, Minneapolis, MN, United States of America; 2 Cold Spring Harbor Laboratory, Cold Spring Harbor, New York, New York, United States of America; 3 Molecular Dermatology, St John’s Institute of Dermatology, King’s College, London, England; 4 Department of Paediatrics, University of Oxford, Oxford, United Kingdom; 5 Department of Biomedicine, Basel University Children’s Hospital, University of Basel, Basel, Switzerland; 6 Department of Biosystems Science and Engineering, ETH Zurich, Basel, Switzerland; Chittaranjan National Cancer Institute, INDIA

## Abstract

Progressive immune deficiency of aging is characterized by severe thymic atrophy, contracted T cell repertoire, and poor immune function. p63 is critical for the proliferative potential of embryonic and adult stem cells, as well as thymic epithelial cells (TECs). Because p63 null mice experience rapid post-natal lethality due to epidermal and limb morphogenesis defects, studies to define a role for p63 expression in TEC biology focused on embryonic thymus development and *in vitro* experiments. Since post-natal thymic stromal development and function differs from that of the embryo, we assessed the impact of lineage-restricted p63 loss on pre- and post-natal murine TEC function by generating mice with a loss of p63 function targeted to TEC, termed p63^TECko^ mice. In adult p63^TECko^ mice, severe thymic hypoplasia was observed with a lack in a discernable segregation into medullary and cortical compartments and peripheral T cell lymphopenia. This profound thymic defect was seen in both neonatal as well as embryonic p63^TECko^ mice. In addition to TECs, p63 also plays in important role in the development of stratified epithelium of the skin; lack of p63 results in defects in skin epidermal stratification and differentiation. Interestingly, all adult p63^TECko^ mice lacked hair follicles despite having normal p63 expression in the skin. Together our results show a critical role of TEC p63 in thymic development and maintenance and show that p63 expression is critical for hair follicle formation.

## Introduction

The thymus is essential for the growth, differentiation and selection of T cells whereby thymic epithelial cells (TECs), fibroblasts, B cells and hematopoietic cells constitute its major stromal cell types enabling normal thymopoiesis to occur [1]. Distinct stages in the intrathymic development of T cells, a.k.a. thymocytes, are distinguished by different cell surface markers including the differential expression of CD4 and CD8. The most immature thymocytes are characterized by a CD4^−^CD8^−^ (double-negative, DN) phenotype and yet lack the expression of a complete T cell antigen receptor (TCR). DN thymocytes are resident in the thymic cortex where they differentiate into more mature CD4^+^CD8^+^ (double-positive, DP) thymocytes that eventually express a TCR [2] and thus undergo in sequential fashion first positive selection and then a first wave of negative thymocyte selection [3–5]. Thymocytes that have successfully passed these two selection steps access the thymus medulla where a second wave of negative selection either eliminates cells with a high TCR specificity or deviates their development to adopt a T regulatory cell fate. Post-selection, mature thymocytes have either a CD4+CD8- or CD4-CD8+ single positive (SP) phenotype and after additional maturation are exported from the medulla to the periphery [2].

The p63 transcription factor is a member of the p53 gene family [6] and exerts pleiotropic functions, including cell proliferation, survival, apoptosis, differentiation, senescence, and aging [7,8]. The *p63* locus architecture has two promoters, and its transcripts are characteristically subject to alternative splicing generating transactivating (TA-p63) or dominant negative (ΔN-p63) isoforms [9]. ΔN-p63 isoforms are most abundant in mature proliferating epithelia and dramatically downregulated upon differentiation and growth arrest [10]. ΔN-p63 is expressed in both cortical (c) TEC and medullary (m) TEC [11]. p63 is also critically important for skin development and maintenance and is predominantly found in the bulge of the hair follicle and to a lesser extent in the interfollicular epidermis and hair bulb [12,13].

A role for p63 in TEC biology has been suggested in mice rendered p63 deficient as these animals demonstrated at birth a severely reduced thymus size likely secondary to functional deficiencies of its non-hematopoietic stromal compartment; a supposition that correlates with an increased rate of TECs apoptosis in newborn mice [12,14]. Because mice germ-line deficient in p63 expression die early after birth, quantitative and qualitative studies concerning a role of p63 expression in TEC function and maintenance beyond the neonatal period were not possible [12]. To circumvent this limitation, we generated mice with a TEC-targeted loss of *p63* expression to answer the question whether p63 expression is essential for postnatal TEC biology. We report here on the effects of p63 deletion in both the thymus and skin.

## Materials and methods

### Mice

Mice were kept under specific pathogen-free conditions and used according to federal and institutional regulations. To generate p63-deficient TECs, mice with a conditional *p63* allele [15] (p63 fl/fl) were crossed to transgenic mice expressing the Cre recombinase under the FoxN1 promoter that drives expression in TECs and skin epithelial cells [16] (both of which are on the C57BL/6 (B6) background, and provided by a co-author, AAM). In mice that are heterozygous for Cre, p63 is deleted in TECs; the p63^+/+^ that are Cre^-^ express p63 in TECs. Mice that were Cre^+^ were considered p63^TECko^; mice that were Cre^-^ were considered p63^+/+^ and are denoted as such. To determine which mice had TEC p63 deficiency in the embryonic experiments, p63^TECko^ mice were genotyped using DNA from tail clippings on embryonic day 15.5 (E15.5) (day 1 is day of gestation), as previously described [15]. All murine studies were conducted under a protocol approved by the University of Minnesota IACUC (protocol 2104-39043A). As outlined in the IACUC protocol, mice were handled humanely and euthanized using the aforementioned University of Minnesota IACUC approved cervical dislocation method to minimize suffering.

### Flow cytometry and cell suspension

Mouse thymus, spleen, and lymph node (LN) were processed into single cell suspensions and analyzed by flow cytometry. Dead cells were stained by a fixable viability dye conjugated to eFluor780 (eBioscience). The following antibodies were purchased from BD biosciences: The following antibodies were purchased from eBioscience: CD3 (145-2C11), CD4 (GK1.5), CD11c (N418), and CD25 (PC61.5). The following antibodies were purchased from BioLegend: CD8 (53–6.7), CD11b (M1/70), CD44 (IM7), CD45 (30-F11), CD62L (MEL-14), NK-1.1 (PK136), Ly-51 (6C3), and UEA-1 were purchased from Vector Laboratories. Anti-activated Caspase-3 antibody (C92-605) was purchased from BD Biosciences. Flow cytometric analysis and cell sorting were performed (FACSAria) using FACSFortessa (BD Biosciences) and FlowJo software (Tree Star).

### BrdU staining

Mice were injected twice intraperitoneally with 1mg (100 μl/mouse) Bromodeoxyuridine (BrdU, BD Bioscience) within 4 hours. One day later, BrdU incorporation was analysed by flow cytometry using the BrdU Flow Kit according to manufacturer’s protocol (BD Bioscience).

### Immunohistochemistry

Intact thymi embedded in optimum cutting temperature (OCT) compound (Sakura, Tokyo, Japan) were snap-frozen in liquid nitrogen and stored at −80°C. Cryosections (7 μm) were fixed by air-drying overnight, blocking with 10% normal horse serum/PBS (Jackson Immunoresearch Laboratories, West Grove, PA), and staining with rabbit-anti-mouse cytokeratin-5 (K5) antibody (Covance, Berkeley, CA) plus a cychrome-5-conjugated goat-anti-rabbit antibody (Invitrogen) and biotinylated mouse-anti-mouse K18 (Progen Biotechnik, Heidelberg, Germany) plus Alexa Fluor 555-conjugated streptavidin (Invitrogen), p63 (4A4, Santa Cruz Biotechnology). Sections were mounted under a cover slip with 4,6-diamidino-2-phenylindole anti-fade solution (Invitrogen) and imaged on the following day at room temperature using an Olympus FluoView 500 Confocal Scanning Laser Microscope (Olympus, Center Valley, PA). Immunofluorescence for Cre+ and Cre- mice.

Immunofluorescent staining was performed on unaffected and affected skin and thymus samples. Affected skin was identified as having no hair or patchy hair. Unfixed tissue was prepared for sectioning by freezing in optimal cutting temperature (OCT, Sakura Finetek USA, Torrance, CA). Tissue sections were cut at 6 μm on a cryostat and placed on positively charged glass slides. Sections were fixed in room temperature acetone for 5 minutes, rehydrated in 1x PBS, permeabilized with 0.2% Triton X for 10 minutes followed by blocking with 10% normal donkey serum for 1 hour (Jackson Immunoresearch, West Grove, PA). Primary antibodies p63 (Boster Biological, Pleasanton, CA, 1:200), Keratin 5 and Loricrin (Biolegend, San Diego, CA, 1:500, 1:200), Ki67 (Abcam, Burlingame, CA, 1:200), CK18 (LifeSpan Biosciences, Seattle, WA 1:200), and Epcam1 Fitc (eBioscience, San Diego, CA 1:100) were applied for 1.5 hours. Corresponding secondary antibodies donkey anti-rabbit cy3 (Jackson Immunoresearch, West Grove, PA 1:500), and Alexa Fluor 488 goat anti-chicken, Alexa Fluor 488 goat anti-rat (Molecular Probes, Eugene, OR, 1:800) were used. All incubations were done at room temperature. Slides were cover slipped with hard set DAPI, 4,6-diamidino-2-phenylindole, (Vector Labs, Burlingame, CA) and examined by confocal fluorescence microscopy (Olympus BX61, Olympus Optical, Tokyo, Japan).

### TEC isolation

TEC single-cell suspension were prepared and sorted based on CD45^−^EpCAM^+^ of cTECs (UEA^−^1-Ly51) and mTECs(UEA-1^+^Ly51^+^I-A^b+^) as previously described [17]. Dead cells were excluded upon a fixable eFluor 780 viability dye staining. UEA-1 and CD45 (30-F11) were purchased from Vector Laboratories and BD Biosciences, respectively. The following Abs were purchased from eBioscience: CD16/32 (93) and MHC class II (AF6-120.1). The following Abs were purchased from BioLegend:CD326 (Ep-Cam, G8.8) and Ly-51 (6C3). FACS data were collected using BD LSR flow cytometer and were analyzed using FlowJo version 10 (Tree Star).

### Statistical analysis

Statistical analysis was performed using Student *t* test (unpaired, two-tailed). Probability values were classified into four categories: *p* > 0.05 (NS), *0.05 ≥ *p* > 0.01, **0.01 ≥ *p* > 0.001, ***p ≤ 0.0001, and **** 0.0001 ≤p ≤0.001.

## Results

### Conditional p63 deletion results in catastrophic thymic hypoplasia

**Fig 1:**
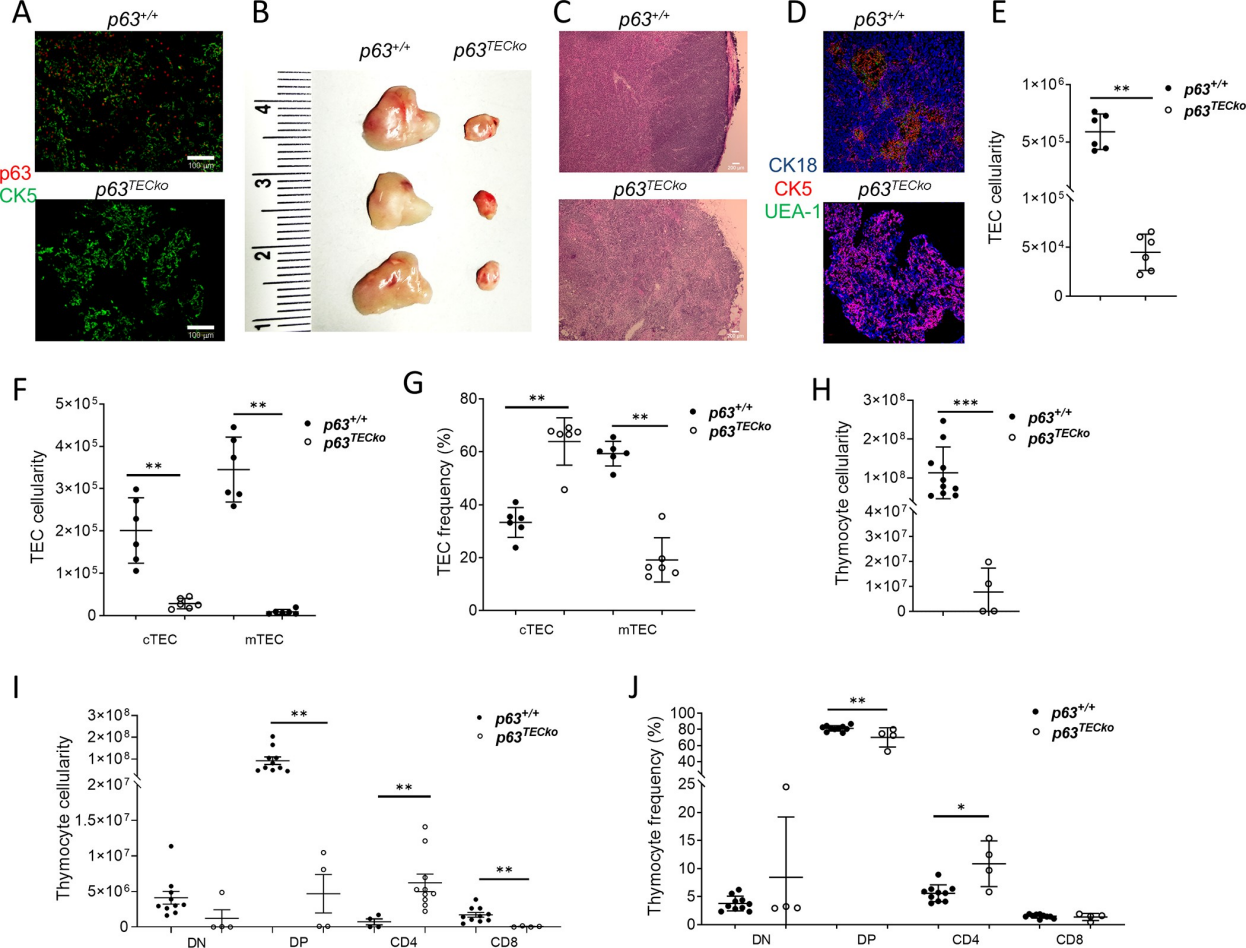
p63^TECko^ mice have profound thymic hypoplasia. Fig 1A shows absence of p63 in p63^TECko^ TECs. Fig 1B shows the thymus size of both p63^+/+^and p63^TECko^ adult mice. Fig 1C shows H and E staining. The scale bars are 50 μm. Fig 1D shows CK18 (blue), CK5 (red) and UEA-1 (green) staining. Absolute numbers of TEC (1E and p = 0.0022), of TEC subsets (1F; cTEC, p = 0.0022; mTEC, p = 0.0022) and frequency of TEC subsets (1G; cTEC, p = 0.0022; mTEC, p = 0.0022) based on (CD45^−^EpCAM^+^ of cTECs (Ly51^+^UEA−1^-^) and mTECs (Ly51^-^UEA-1^+^I-A^b+^) are shown. Flow cytometric analysis for the cell-surface expression of CD4 and CD8 on thymocytes isolated from p63^TECko^ and littermate control mice. Numbers denote absolute number of cells of total thymocytes (1H; p = 0.0022) and subsets (1I; DP, p = 0.0011; CD4, p = 0.0077; CD8, p = 0.0022), and frequency of the subtypes (1J; DP, p = 0.0077; CD4, p = 0.0297). P values are shown.

To establish a specific role for p63 in TEC development and function, we generated and analyzed mice with a conditional loss of *p63* targeted to all TEC (designated p63^TECko^) due to a deletion of *p63* exons 5, 6 and 7 which alters the reading frame and thus affects the function of any protein should it be generated [15]. In embryos at day 15.5 of development (E15.5) p63^TECko^, p63 was undetectable in TECs using immunohistochemistry and confocal microscopy (Fig 1A), validating our experimental model.

We first sought to assess the effect of p63 deletion on TEC cellularity and architectural organization in 2–4-month-old mice. The thymus p63^TECko^ was significantly smaller in size (Fig 1B) and displayed an abnormal histological architecture in H&E stains with no discernible demarcation between the cortex and medulla (Fig 1C). TECs can be classified based on their positional, structural, antigenic, and functional characteristics either as cTECs (flow cytometrically identified as Ly51^+^UEA1^-^) or as mTECs (Ly51^-^UEA1^+^) [18]. The architectural organization and composition of the TEC scaffold in cortex and medulla was assessed by immunohistology using anti-keratin antibodies directed at keratin (K)18 for cTECs and K5 for mTECs as well as reactivity to *Ulex europaeus* agglutinin 1 (UEA-1), a lectin that binds to mTECs [18]. A detailed investigation of the epithelial compartment showed that p63^TECko^ had a disorganized structure that lacked individual and well demarcated medullary islands (Fig 1D) a finding that correlated with a loss of a distinct cortico-medullary junction. Fewer TECs were detected in mutant when compared to p63^+/+^ (Fig 1E; p = 0.0022). This reduction affected both the cTEC (p = 0.0022) and mTEC population (p = 0.0022) (Figs 1F, 1G and S1) and correlates with reduced proliferative potential of TECs in p63^TECko^ mice (S2 Fig). The ratio of cortical to medullary TEC was changed in p63^TECko^ mice secondary to a higher frequency of cTEC (p = 0.0022) relative to mTEC (p = 0.0022) when compared to p63^+/+^ mice.

Total thymocyte numbers were also significantly decreased (p = 0.0022) and this was seen in all subsets in p63^TECko^ mice (Fig 1H–1J). The decrease in cell number also translated to significant decreases in frequencies of DP (p = 0.0077), and CD4 SP (p = 0.0297) but not the CD8 SP (p = 0.9731) or DN (p = 0.9527) subsets in the p63^TECko^ mice (Fig 1I and 1J), as well as significant decreases in the frequency of FoxP3^+^ Treg (S3 Fig; p <0.0001). Taken together, our data shows that p63 expression in TECs is required for the establishment of a normal thymic microenvironment, function and sustained integrity of the thymus.

### P63 deletion in embryonic mice results in decreased TECs and immature thymocytes

**Fig 2:**
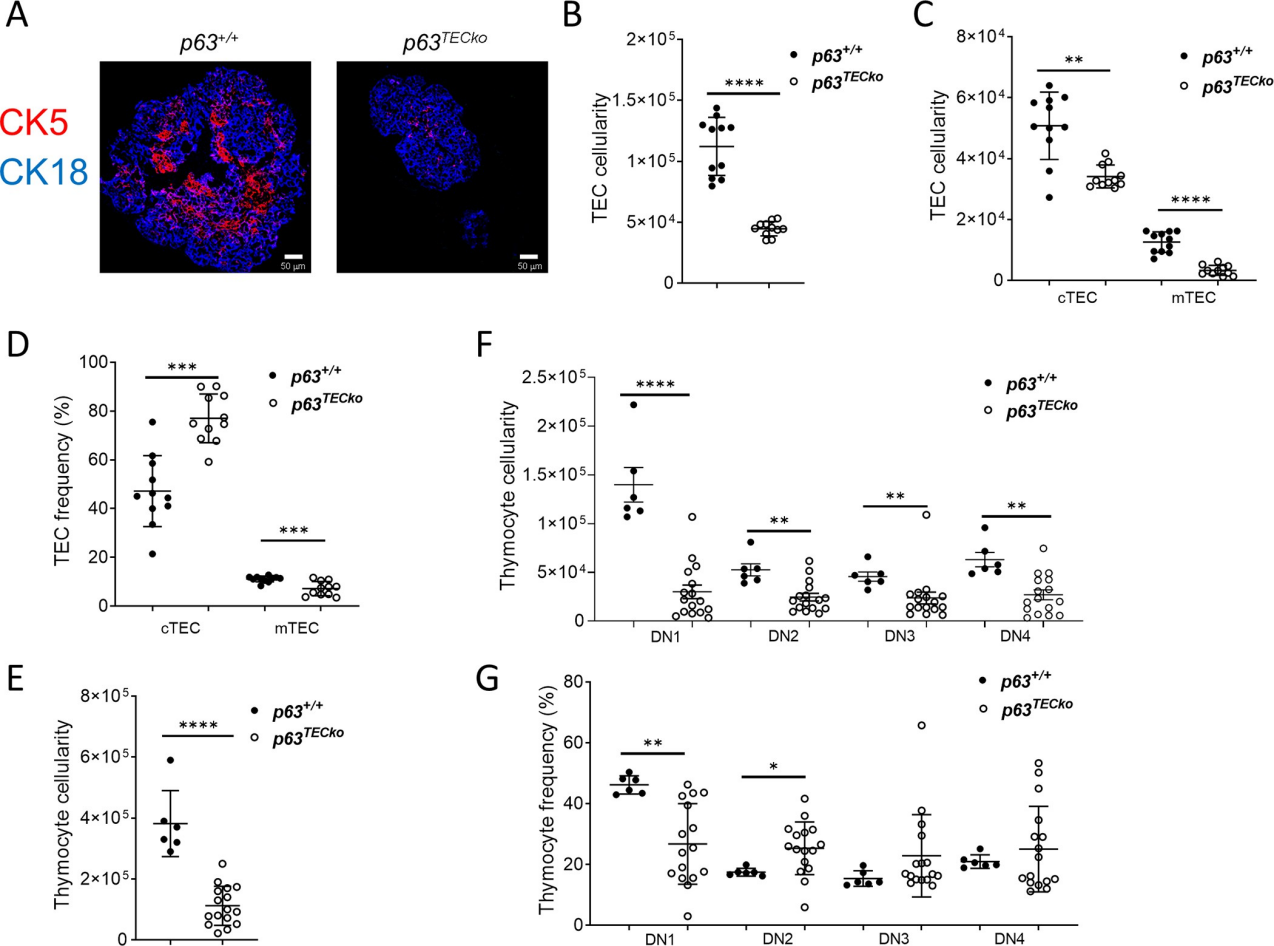
p63^TECko^ mice have decreased TECs and DN1 cells in embryonic mice. Fig 2A shows CK18 (blue), CK5 (red) staining. The scale bars are 50 μm. Absolute TEC cellularity (2B, p<0.0001) numbers of TEC subsets (2C; cTEC, p = .0014; mTEC, p<0.0001) and frequency of TEC subsets (2D; cTEC,p = 0.001;mTEC,p = 0.008)) based on (CD45^−^EpCAM^+^ of cTECs (Ly51^+^UEA−1^-^) and mTECs(Ly51^-^UEA-1^+^I-A^b+^) are shown. Flow cytometric analysis for the cell-surface expression of CD44 and CD25 on thymocytes isolated from p63^TECko^ and p63^+/+^mice. Thymocyte cellularity of total thymocytes (2E, p = <0.0001) and DN subsets (2F; DN1, p = 0.0004; DN2, p = 0.0151; DN3, p = 0.0243; DN4, p = 0.0036) frequency of DN subsets (2G; DN1, p<0.0001). This experiment was performed 2 times with at least 3 mice per group. P values are shown.

Due to the profound defect in adult mice, we wanted to see if we could pinpoint when the thymic defect occurred. In order to accomplish this, E15.5 thymic lobes were examined. As shown in Fig 2, immunohistochemistry showed that the frequencies of mTECs (K5^+^) but not cTECs (K18^+^) were decreased in p63^TECko^ thymus compared to p63^+/+^ tissue sections (Fig 2A). At E15.5, separate medullary islands had formed in littermate embryos whereas the morphology of the thymic medulla in p63^TECko^ mice appeared to be largely positioned in the center of the lobe as a single large aggregate mTEC (Fig 2A). The absolute number of TECs (p<0.0001) was significantly decreased in p63^TECko^ mice when compared to p63^+/+^ (Fig 2B). This reduction affected both the cTEC (p = 0.014) and the mTEC (p<0.0001) compartments (Fig 2C). Additionally, the frequencies of cTECs were elevated in the p63^TECko^ mice (P = 0.0001) whereas the frequencies of mTECs were lower in the p63^TECko^ mice (p = 0.0008) compared to p63^+/+^ (Fig 2D). To examine the functional consequences of loss of p63 expression in TECs, we examined thymopoiesis of E15.5 p63^TECko^ mice using single cell suspensions and analyzed the thymus for CD44 and CD25. As shown in Fig 2E, total thymic cellularity was also decreased in p63^TECko^ mice. Immature thymocyte population examined were double negative (DN) cells that are characterized by CD44 and CD25 expression: DN1 (CD25^-^CD44^+^), DN2 (CD25^+^CD44^+^), DN3 (CD25^+^CD44^-^) and DN4 cells (or pre-DP cells; CD25^-^CD44^-^). In assessing the different stages of DN cells, there was a significant decrease in the cellularity of all DN stages (Fig 2F) in p63^TECko^. This decrease in cell number also correlated with a decrease in DN1 frequency in the p63^TECko^ mice (p<0.0001; Fig 2G). Taken together, this data shows that decreased TEC numbers and altered thymus architecture affect mainly the most immature DN1 stage of thymocyte development but not yet the more mature DN subsets in embryonic p63^TECko^ mice.

### P63 deletion in postnatal TEC compromises thymus function

**Fig 3:**
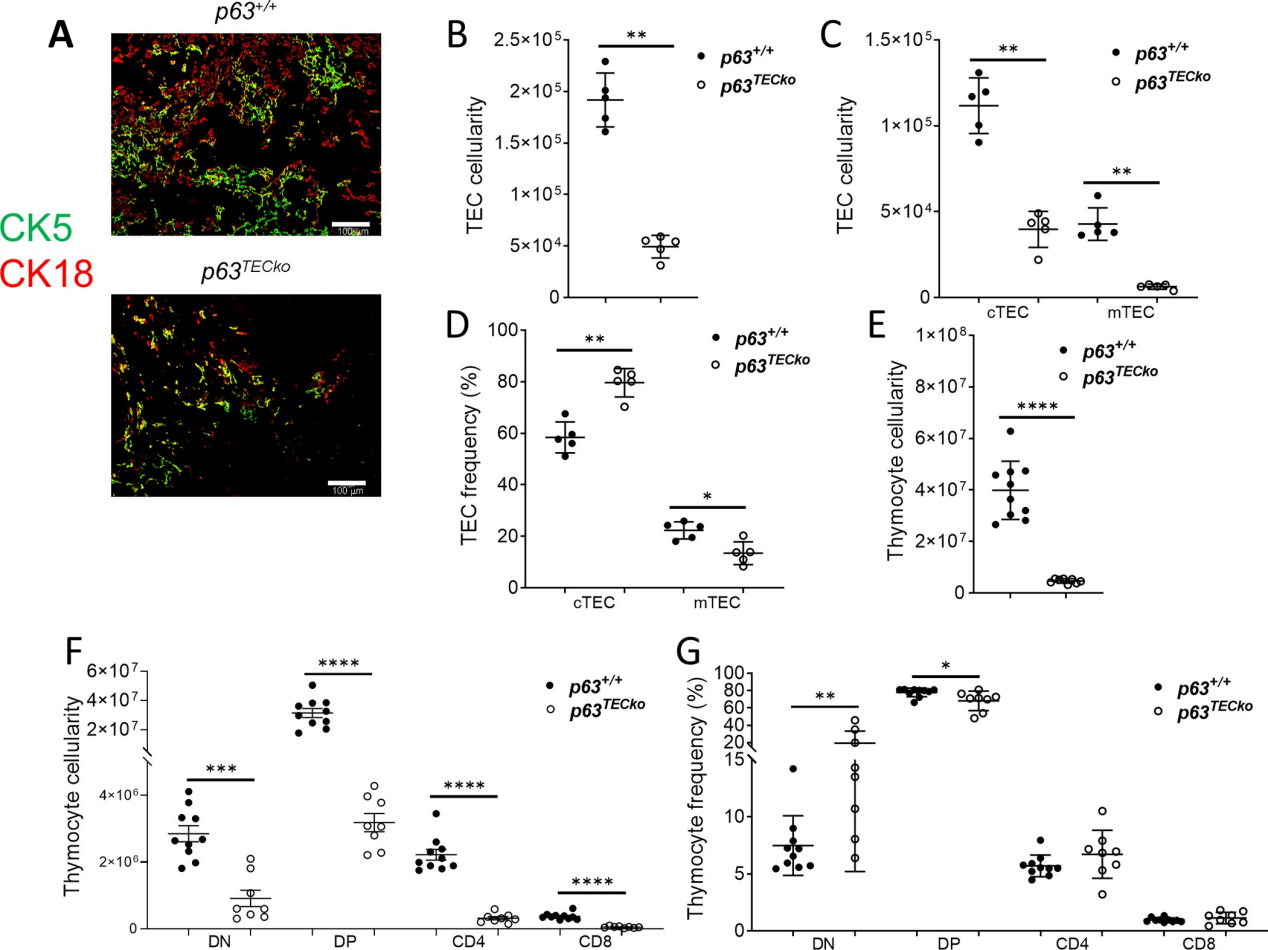
Newborn p63^TECko^ mice have severe thymic hypoplasia. Fig 3A shows abnormal staining of the thymus in p63^TECko^ mice in the CK5 (green) and CK18 (red) compartments. The scale bars are 100 μm. Absolute TEC (3B, p = 0.0079), numbers of TEC subsets (3C; cTEC, p = 0.0079; mTEC, p = 0.0079) and frequency of TEC subsets (3D; cTEC, p = 0.0079; mTEC, p = 0.0317) based on (CD45^−^EpCAM^+^ of cTECs (Ly51^+^UEA−1^-^) and mTECs(Ly51^-^UEA-1^+^I-A^b+^) are shown. Flow cytometric analysis for the cell-surface expression of CD4 and CD8 on thymocytes isolated from p63^TECko^ and p63^+/+^mice. Numbers denote absolute number of total thymocytes (3E, p<0.0001) and subsets (3F; DN, p = 0.0002; DP, p<0.0001; CD4, p<0.0001; CD8, p<0.0001) and frequencies of the subtypes (3G, DN, p = 0.0062; DP, p = 0.0328). This experiment was performed 2 times with at least 3 mice per group. P values are shown.

We next investigated the structural and functional consequences of a lack of p63 expression in TECs in postnatal mice. The thymus architecture in the p63^TECko^ mice was very altered based on CK5 and CK18 staining (Fig 3A); the p63^TECko^ mice had minimal staining of both the cortex and medulla due to its small size. In p63^TECko^ newborn mice, total TECs (p = 0.0079), as well as both cTECs (p = 0.0079) and mTECs (p = 0.0079), (Fig 3B and 3C respectively) were significantly decreased. Similar to E15.5 p63^TECko^ mice, the frequency of cTEC was higher (p = 0.0079) and mTEC (p = 0.0317) was lower in p63^TECko^ mice (Fig 3D). The loss of TECs in p63^TECko^ had major deleterious effects on thymocyte development. Total thymus cellularity was significantly decreased in p63^TECko^ newborn mice as compared to p63^+/+^ (Fig 3E). This reduction correlated with a marked decrease of all major thymocyte subpopulations (Fig 3F). The frequency of DP cells was significantly lower in p63^TECko^ newborn mice (p = 0.0328); however, the predominant population is DP cells in the p63^+/+^ as well as the p63^TECko^ newborn mice (Fig 3G). Collectively, this data shows that by the newborn stage, p63 deficiency in TECs has a profound defect in not only thymic architecture, but also thymocyte development.

### The naïve, but not memory T-cell subset, is reduced in secondary lymphoid tissues of p63^TECko^ mice and contracts over time in postnatal mice

**Fig 4:**
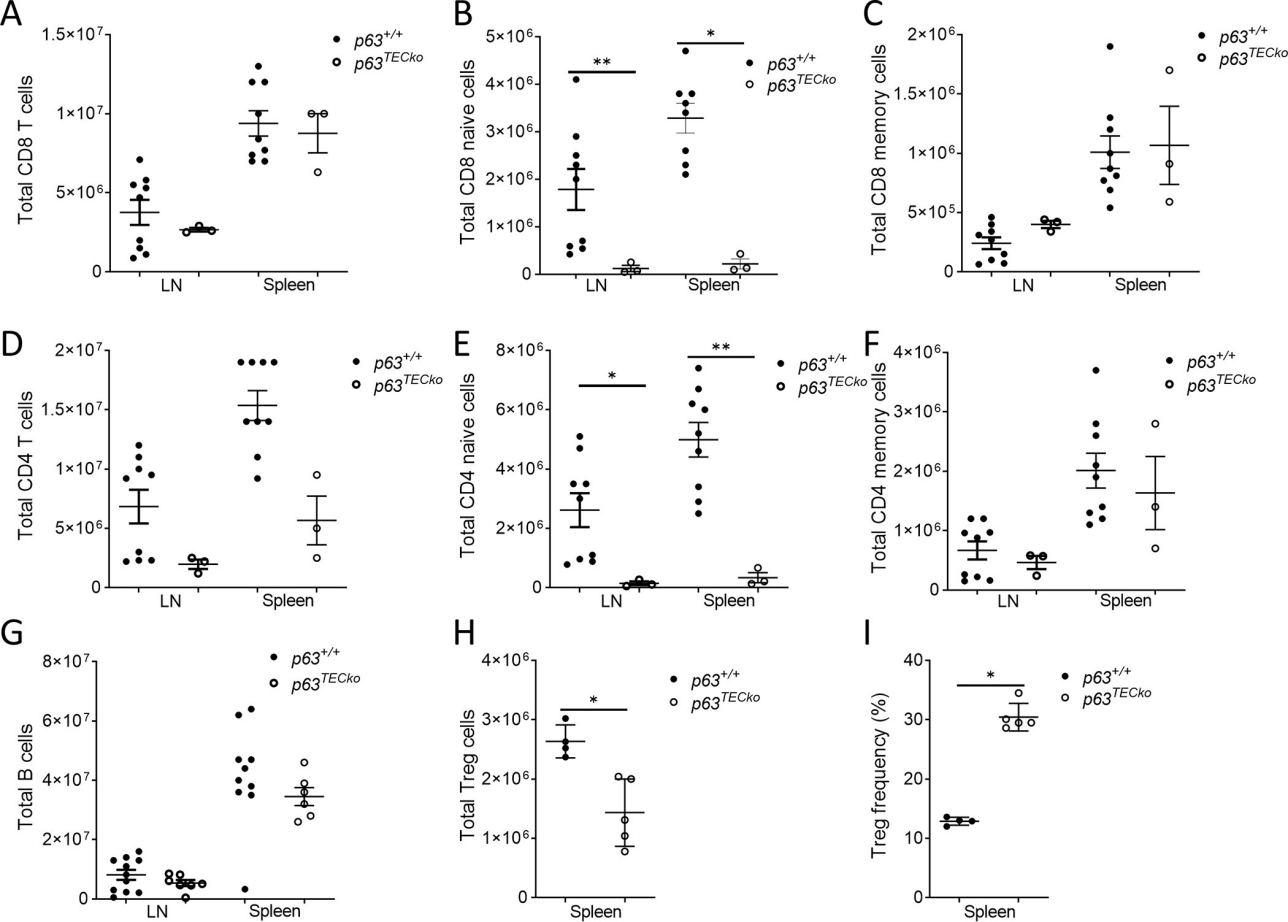
The peripheral T cell compartment shows decreased naïve cells in p63^TECko^ mice. Flow cytometric analysis for the cell-surface expression of CD4 and CD8 splenocytes and lymphocytes were isolated p63^TECko^ and littermate controls. Regulatory T cells were defined as CD4^+^CD25^bright^ and FoxP3^+^. Numbers denote absolute number of cells. (Total CD8 (4A), CD8 naive (4B; LN, p = 0.0573; spleen, p = 0.0003), CD8 memory (4C), total CD4(4D; spleen, p = 0.0031), CD4 naive (4E; LN, p = .0370; spleen, p = 0.0013), CD4 memory (4F), B cells (4G), Treg (4H, p = 0.0159) and frequency of Treg (4I, p = 0.0159). This experiment was done at least twice with at least three mice per group. P values are shown.

Previous studies were unable to examine the peripheral T-cell compartment due to the rapid mortality of nonconditional p63 deficient mice that die within hours after birth [12]. To assess the peripheral compartment, spleen and lymph node T-cell constituency were quantified in 2-month-old mice. In order to address peripheral homeostasis, we further categorized CD4 and CD8 T-cells into naïve (CD62L+CD44-) and memory (CD62Llo or CD62lhi, CD44hi) subsets. Despite severe thymic atrophy by the newborn stage in p63^TECko^ mice, there was no difference between the groups in total CD8 cell number in either the spleen or lymph nodes (Fig 4A). There was a 10-fold reduction in CD8 naïve T-cells in the spleen (Fig 4B, p = 0.0003) with no differences in the CD8 memory compartment (Fig 4C) in p63^TECko^ mice. In the CD4 compartment of p63^TECko^ mice, there were significantly fewer CD4 T-cells in the spleen (p = 0.0031), but not in lymph nodes (Fig 4D). Naïve CD4 T-cells were significantly decreased by 10-fold in both the spleen and lymph node of p63^TECko^ mice (Fig 4E). As seen for CD8 T-cells in p63^TECko^ mice, there was not a difference in memory CD4 T-cells (Fig 4F) between the groups. Of the other populations examined, TEC p63 deficiency did not alter the absolute numbers of splenic B-cells in the lymph node, while slightly reducing absolute splenic B-cells (Fig 4G). The number of regulatory T cells (Treg; defined as CD4+25bright and FoxP3+) as well as the frequency of Tregs was reduced in p63^TECko^ mice when compared to p63^+/+^ (Fig 4H and 4I I; P = 0.0159, P = 0.0159, respectively). This data shows that p63 expression is required for peripheral maintenance of naïve T cells but not required for the persistence of memory T cells.

### p63^TECko^ mice have abnormal patches of skin and absent hair follicles

**Fig 5:**
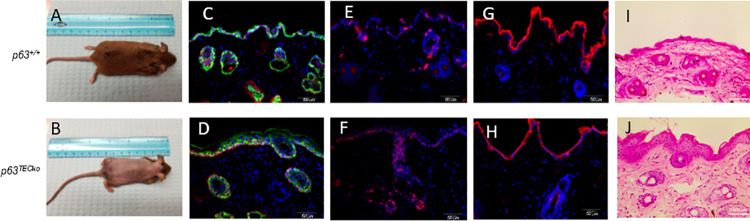
p63^TECko^ mice have abnormal patches of skin and absent hair follicles. p63^TECko^ mice are hairless compared to p63^+/+^ mice (5B and 5A respectively). Fig 5C and 5D shows Cytokeratin 5 (green), p63 (red) and DAPI (blue); Fig 5E and 5F shows Epcam1 (green), Ki67 (red) and DAPI (blue); Fig 5G and 5H shows Loricrin (red) and DAPI (blue); H and E staining of p63^TECko^ mice show the absence of hair follicles (Fig 5I and 5J). The scale bars are 50 μm in 5C to 5H, and 1000 μm in 5I and 5J. This experiment was performed at least twice with at least three mice per group each time.

It is known that p63 is expressed in keratinocytes [10] and p63 knockout mice have a complete loss of stratified epithelium [12]. In addition, the epidermis in p63 knockout mice is non-continuous and fragmented compared to p63^+/+^ [12]. FOXN1 deficiency results in a “nude” mouse. These mice are hairless not due to a lack of hair follicles but because these follicles have an absence of free sulfhydryl groups in the mid-follicle region and the hair twists and coils [19]. Although *FOXN1* is not expressed in epithelial stem cells of the skin, *FOXN1* expression is important in terminal differentiation of epithelium [20], leaving the possibility that skin lesions would occur as a result of terminally differentiated stratified epithelium loss. In p63^TECko^ mice, there were no differences in skin at E15.5 or newborn stage compared to p63^+/+^ (S4 Fig). However, by 2 months of age, surprisingly, 100% of p63^TECko^ mice had abnormal patches of skin that showed no hair (Fig 5A and 5B). We next wanted to assess p63 protein expression between the groups. As shown in Fig 5C and 5D, p63 (shown in red) is abundant in both littermate control and p63^TECko^ skin epidermis. In addition, the skin of p63^TECko^ mice shows continuous epidermis (Fig 5C). Neither p63^TECko^ skin or p63^+/+^ skin expressed EpCAM (Fig 5E and 5F). There were no differences in other skin antigens between p63^TECko^ mice and their p63^+/+^ including Cytokeratin 5 (Fig 5C and 5D), Ki67 (Fig 5E and 5F) or loricrin, a terminal differentiation marker in keratinocytes (Fig 5G and 5H) [14]. In assessing the differences based on hematoxylin and eosin staining, p63^TECko^ mice showed that there was an absence of hair follicles which are lined typically with stratified epithelium, compared to p63^+/+^ (Fig 5I and 5J). Taken together, these data show that the hair follicles in p63^TECko^ mice are the only areas profoundly affected in the skin. This hair loss is due to the absence of hair follicles and not because of a loss of sulfhydryl groups as in Nude mice.

## Discussion

Our data has shown that loss of p63 expression in TECs causes catastrophic loss of thymocytes. Decreases in TECs did not result in abnormal numbers of thymocytes at E15.5 in p63^TECko^ mice. However, in post-natal mice, there was significant thymic hypoplasia in the thymocyte compartment in all subsets. These data indicate that p63 function is critical in TECs to ensure normal thymocyte differentiation. Moreover, normal p63 expression is also required for normal T cell maintenance.

In p63^TECko^ mice, 100% of mice by 2 months of age have abnormal patches of skin suggesting that different regulatory elements were responsible for FOXN1 expression and hence p63 expression in these mice. p63 is found in the interfollicular epidermal stem cells and is important for stem cell proliferation [21]. The syndrome called AEC (ankyloblepharon-ectodermal defects-clefting) is an autosomal dominant ectodermal dysplasia disorder caused by mutations in the transcription factor p63 which can clinically manifest in alopecia or sparse hair [22]. We hypothesize that despite p63 being expressed in the stem cells, it was most likely not expressed in the hair follicle stem cells or key progeny, resulting in absence of hair follicles in p63^TECko^ mice.

There have been multiple models which explored the role of p63 in development. In the complete p63 knockout mice, major defects in limb, craniofacial and epithelial development, a severely abnormal thymus and absence of all squamous epithelium were noted [12]. Koster et al showed that p63 plays a dual role in development: initiating epithelial stratification and maintaining proliferative potential of basal keratinocytes in mature epidermis [10]. This was reinforced by Truong et al who showed that down-regulation of p63 in keratinocytes using specific siRNAs resulted in in severe tissue hypoplasia and inhibited both stratification and differentiation in a cell-autonomous manner in keratinocytes [13]. It is known that disruption of FoxN1 function in mice leads to a profound immune deficiency and a hairless phenotype [23]. FoxN1 deletion in human also results in a catastrophic T cell deficiency, congenital alopecia and nail dystrophy [19]. In a recent study, Larsen and colleagues found a *cis*–regulatory element that was critical for expression of *Foxn1* in TECs but dispensable for expression in hair follicles; in these mice, *Foxn1* expression and function in the hair follicle were unaffected [24].

In p63^TECko^ mice, p63 deficiency was restricted to TECs and had severe thymic hypoplasia. These data emphasized the importance of p63 in TECs resulting in the loss of both TECs and thymocytes, most likely as result of the absence of crosstalk that produces bidirectional signaling between TECs and thymocytes, critical in maintaining the thymic microenvironment [24–28]. Senoo et al showed severe thymic hypoplasia in p63 knockout mice; they also observed that K5 and K8 as well as UEA-1 [25–27] expression patterns were indistinguishable between littermate and p63 knockout thymuses at E15.5 [12]. The authors further proved that the thymic defect occurred as a consequence of p63 deficiency in TECs and not in the lymphoid compartment as Rag2-deficient mice that lack mature B and T cells exhibited normal thymic development when reconstituted with p63 knockout hematopoietic stem cells. Thymus hypoplasia was attributed to loss of proliferative potential of thymic epithelial stem cells and enhanced TEC apoptosis. Our data support and further this point by showing the importance of p63 expression in TECs. Taken together our data suggest that p63 is not only important in TEC integrity, but also in preventing thymic involution and in development and maintenance of hair follicles (see S5 Fig).

## Supporting information

S1 Fig Representative flow cytometry plots are shown for mTEC and cTEC in E15.5 (S1A Fig) and neonate (S1B Fig) p63+/+ and p63TECko mice. mTEC are defined as Ly51^-^UEA1^+^ and cTEC are defined as Ly51^+^UEA−1^-^. Representative flow cytometry plots are shown for thymic CD4 and CD8 expression profiles in E15.5 (S1C Fig) and neonate (S1D Fig) mice.
(TIF)

S2 Fig p63^TECko^ mice have reduced TEC proliferation. Absolute numbers (S2A Fig; cTEC, p = 0.0026; mTEC, p = <0.0001) and frequency (S2B Fig; cTEC, p = 0.0001; mTEC, p = 0.0011) of TEC subsets were reduced in p63^TECko^ compared to p63^+/+^ controls. This reduction in both mTECs and cTECs correlated with reduced proliferative potential of TECs in p63^TECko^ mice (S2C Fig; cTEC, p = 0.0123; mTEC, p = 0.0386), but no change in Caspase-3 expression (S2D Fig; mTEC, p = 0.0264) within TEC subsets. P values are shown.
(TIF)

S3 Fig p63^TECko^ mice have a profound reduction in Treg absolute numbers and frequencies. Total number (3A; p<0.0001) and frequency of FoxP3^+^ Treg (3B; p = 0.0184). P values are shown.
(TIF)

S4 Fig E15.5 and newborn p63^TECko^ mice didn’t show any visible hair loss in skin compared to their p63^+/+^ littermates. Panels A-D shows Cytokeratin 5 (green), p63 (red) and DAPI (blue); Panels E-H show Ki67 (red) and DAPI (blue); Panels I-L shows Loricrin (red) and DAPI (blue); Panels M-P show H and E staining of skin at E15.5 and Newborn timepoints.
(TIF)

S5 Fig Graphical summary of the key differences and similarities in thymic phenotype upon TEC-specific p63 deletion in E15.5 and neonate mice. (TIF)

S1 Data (XLSX)
