# Supplementary figures and images for: P63 targeted deletion under the FOXN1 promoter disrupts pre-and post-natal thymus development, function and maintenance as well as induces severe hair loss
Source: PLoS One. 2022 Jan 25;17(1):e0261770. doi: 10.1371/journal.pone.0261770 (PMC8789144; doi:10.1371/journal.pone.0261770)

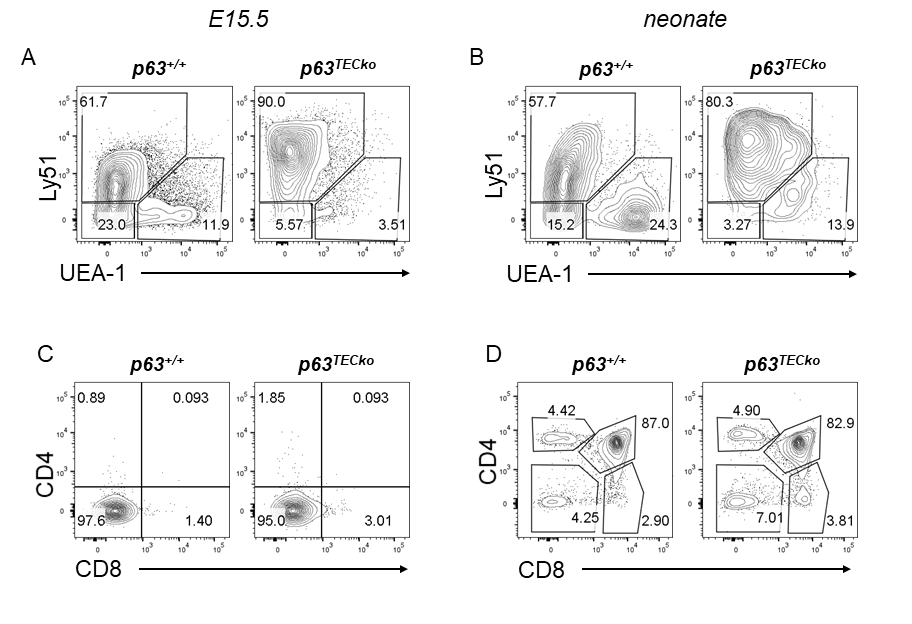

Supplement: S1 Fig — mTEC are defined as Ly51-UEA1+ and cTEC are defined as Ly51+UEA−1-. Representative flow cytometry plots are shown for thymic CD4 and CD8 expression profiles in E15.5 (S1C Fig) and neonate (S1D Fig) mice. (TIF) [file pone.0261770.s001.tif]

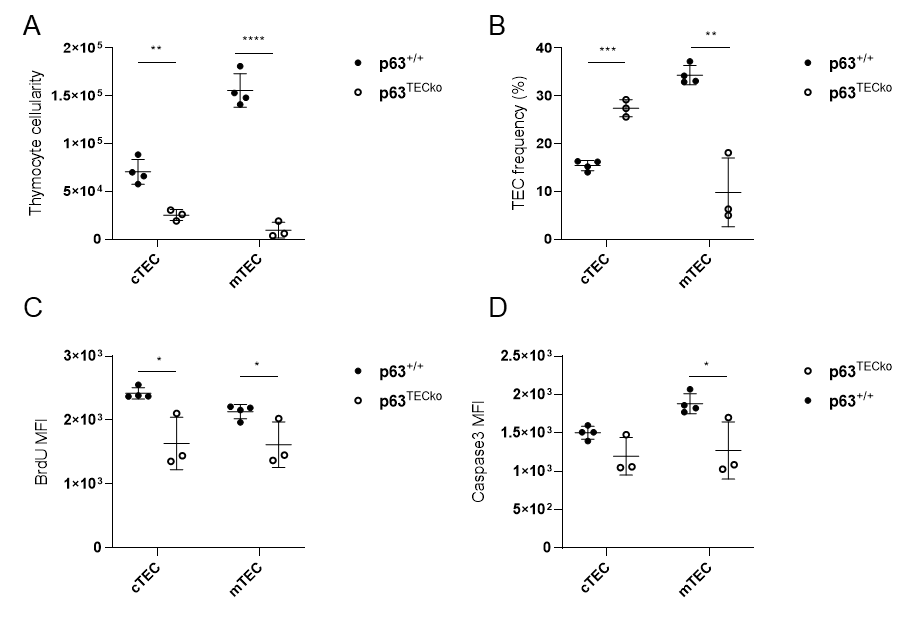

Supplement: S2 Fig — Absolute numbers (S2A Fig; cTEC, p = 0.0026; mTEC, p = <0.0001) and frequency (S2B Fig; cTEC, p = 0.0001; mTEC, p = 0.0011) of TEC subsets were reduced in p63TECko compared to p63+/+ controls. This reduction in both mTECs and cTECs correlated with reduced proliferative potential of TECs in p63TECko mice (S2C Fig; cTEC, p = 0.0123; mTEC, p = 0.0386), but no change in Caspase-3 expression (S2D Fig; mTEC, p = 0.0264) within TEC subsets. P values are shown. (TIF) [file pone.0261770.s002.tif]

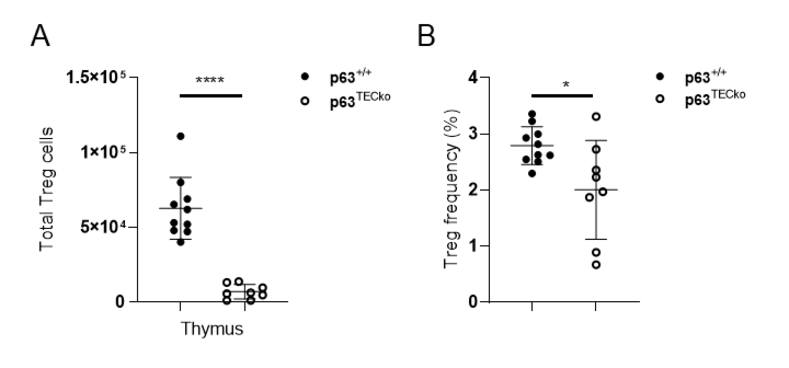

Supplement: S3 Fig — Total number (3A; p<0.0001) and frequency of FoxP3+ Treg (3B; p = 0.0184). P values are shown. (TIF) [file pone.0261770.s003.tif]

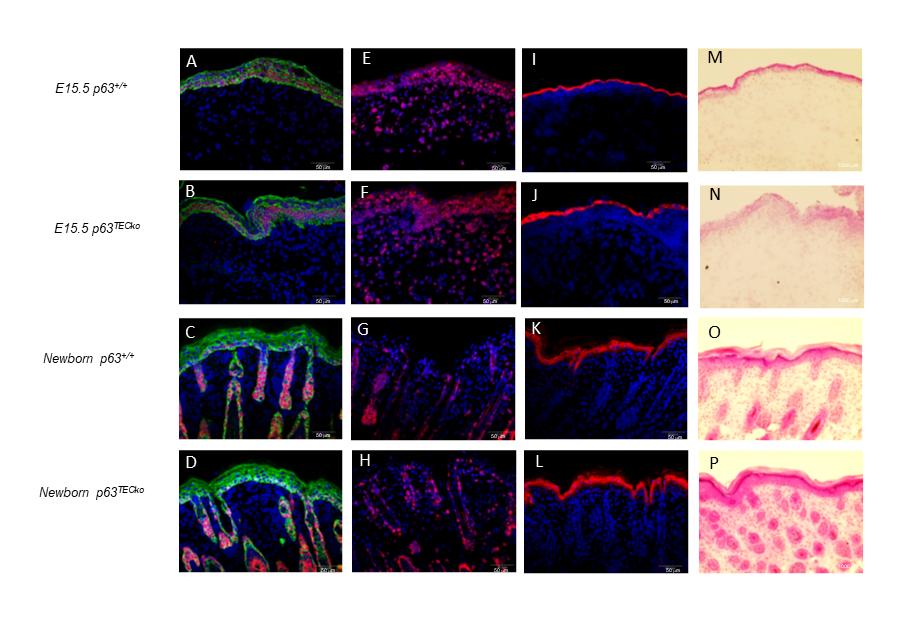

Supplement: S4 Fig — Panels A-D shows Cytokeratin 5 (green), p63 (red) and DAPI (blue); Panels E-H show Ki67 (red) and DAPI (blue); Panels I-L shows Loricrin (red) and DAPI (blue); Panels M-P show H and E staining of skin at E15.5 and Newborn timepoints. (TIF) [file pone.0261770.s004.tif]

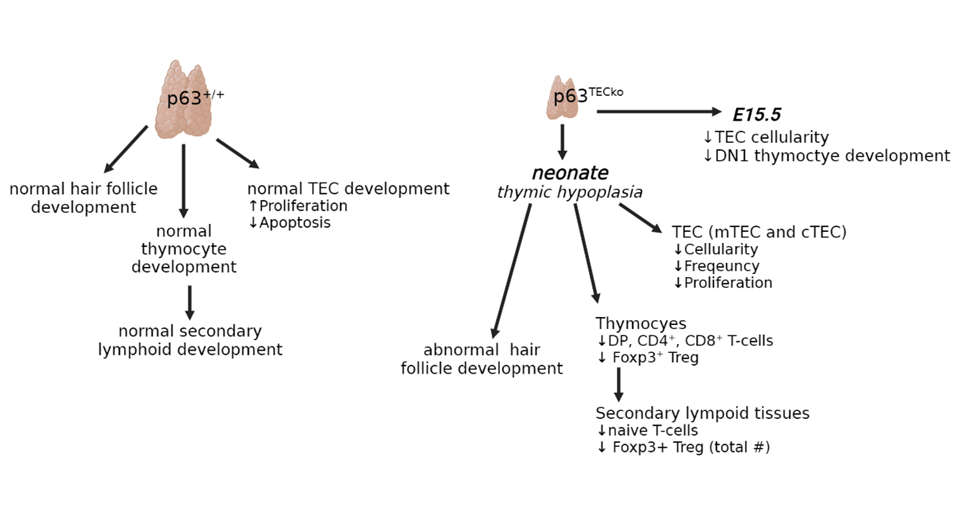

Supplement: S5 Fig — (TIF) [file pone.0261770.s005.tif]
